# Intra-abdominal Mass, Obstructive Jaundice, and Eosinophilia

**DOI:** 10.2478/rir-2022-0015

**Published:** 2022-07-06

**Authors:** Li Wang, Guizhi Zhang, Wenjie Zheng, Xinping Tian, Mengtao Li, Xiaofeng Zeng, Fengchun Zhang

**Affiliations:** 1 Department of Rheumatology and Clinical Immunology, Peking Union Medical College Hospital, Chinese Academy of Medical Sciences and Peking Union Medical College, Key Laboratory of Rheumatology and Clinical Immunology, Ministry of Education, National Clinical Research Center for Dermatologic and Immunologic Diseases, Beijing, 100730, China

**Keywords:** eosinophilic granulomatosis with polyangiitis (EGPA), eosinophils, lymphoma

## Abstract

Eosinophilic granulomatosis with polyangiitis(EGPA) is a systemic vasculitis syndrome associated with eosinophilia, which most commonly involves the lung, skin, cardiovascular, gastrointestinal, renal, and peripheral nervous systems (PNS). We report a case of a 48-year-old man presented as obstructive jaundice caused by intra-abdominal mass, and he also had elevated peripheral eosinophils. The pathological features of the mass included vasculitis and eosinophils infiltration. At first he was diagnosed as EGPA and treated by glucocorticoid and cyclophosphamide. The patient did not get complete response after six months and then the repeat biopsy proved that he had non-Hodgkin's lymphoma.

A 48-year-old male was admitted with the complaint of abdominal pain with a mass for 1 year, and skin and sclera jaundice for 6 months.

One year before admission, he had abdominal pain and noticed an egg-sized, hard mass without tenderness around the navel. He had no fever or other symptoms. He denied any history of asthma or allergic rhinitis. His white blood cell count (13.58 × 10^9^/L) and eosinophil count (4.17 × 10^9^/L, 30.7%) were elevated. An elevated erythrocyte sedimentation rate (ESR) of 31 mm/1 h, and a C-reactive protein (CRP) level of 16.4 mg/L, were also recorded. Blood tests for serum immunoglobulin G4 (IgG4), rheumatoid factor (RF), anti-nuclear antibody (ANA), antineutrophil cytoplasmic antibody (ANCA), antiphospholipid antibodies (APLs), and TB spot were all negative. Computed tomography (CT) scan showed a mesenteric mass. The mass was surgically removed, and pathological examination revealed a small abscess, vasculitis, and infiltration of inflammatory cells, including lymphocytes, plasma cells, eosinophils, and neutrophils.

The patient was diagnosed with systemic vasculitis, probably eosinophilic granulomatosis with polyangiitis (EGPA) based on the pathologic features. Prednisone 60 mg/d and cyclophosphamide (CYC) 100 mg/d were administered orally. Then, he gradually improved. His steroid dose was tapered to 10 mg/d, and CYC was stopped 3 months later.

Six months later, the skin and sclera turned yellowish again. Meanwhile, the patient experienced progressive fatigue and itching, which caused him deep concern. His biochemical test showed elevated total and direct bilirubin, 15.3 mg/dL and 13.8 mg/dL, respectively, with high alkaline phosphatase (ALP) (1289 U/L) and gamma-glutamyltransferase (γ-GT) (1837 U/L). The white blood cell and eosinophil counts were also elevated, amounting to 25.93 × 10^9^/L and 18.84 × 10^9^/L (72.7%), respectively. A repeat CT scan showed a solitary mass embracing the head of the pancreas, ductus choledochus, and duodenum (Figure 1). Percutaneous transhepatic biliary drainage was performed to relieve the jaundice, and a repeat biopsy by endoscopic retrograde cholangiopancreatography (ERCP) suggested non-Hodgkin's lymphoma (NHL).

**Figure 1:**
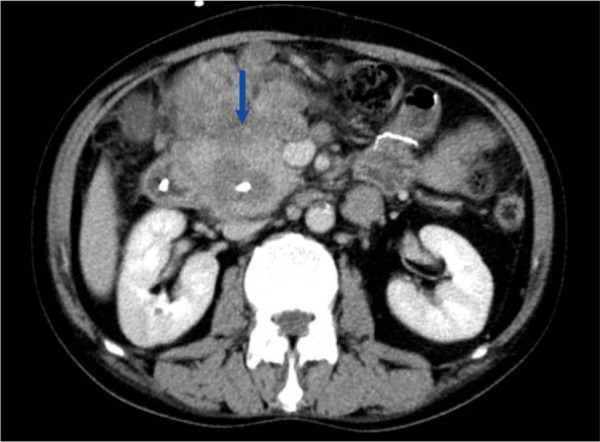
The mesenteric mass wrapped around the head of the pancreas, ductus choledochus, and duodenum. (Blue arrow).

In this case, a middle-aged male had an abdominal mass and obstructive jaundice. The common causes of obstructive jaundice and mass include biliary tract or pancreatic cancer and gallbladder carcinoma. IgG4-related diseases may be rare causes. This patient was suspected of having cancer or carcinoma when he underwent the first operation, but the biopsy did not support these working diagnoses, instead suggesting an inflammatory mass. He also had an increased acute-phase reactant suggestive of systemic inflammatory diseases. Considering his elevated serum acute-phase reactants, high peripheral eosinophil count, being negative for autoantibodies, and normal serum IgG4 level, he was diagnosed with systemic vasculitis, most probably EGPA. He responded well to glucocorticoids and immunosuppressants. However, the patient had jaundice within 6 months, and a repeat biopsy proved that he had NHL.

Hypereosinophilia is caused by various factors, including parasitic infections, allergic disorders, drug hypersensitivity, adrenal insufficiency, and connective tissue diseases such as EGPA, sarcoidosis, IgG4-related disease, and lymphomas. Idiopathic hypereosinophilic syndrome (HES) neoplastic diseases, such as primary (or neoplastic) HES, acute eosinophilic leukemia, systemic mastocytosis, and lymphoma, although rare, may also be implicated and should be differentiated.

EGPA is a major vasculitis syndrome associated with eosinophilia. Patients typically present with asthma or allergic rhinitis as prodromal symptoms, which most commonly involve the lung, skin, cardiovascular, gastrointestinal, renal, and peripheral nervous systems (PNS).^[1]^ Vasculitic symptoms typically develop over the years. Positive ANCA is found in 20–50% of patients with EGPA, and the main pathological features include eosinophilic infiltration, eosinophilia, giant cell vasculitis, and sometimes interstitial and perivascular necrotizing granulomas.^[2]^ The patient had no history of asthma or allergic rhinitis and did not have typical manifestations of vasculitis-related skin or PNS involvement. He was diagnosed with EGPA because of eosinophilic infiltration and vasculitis on biopsy. Furthermore, mesenteric masses are not common in EGPA.

Further pathological examination of the dissected mass showed lymphocytes cells at the peripheral area of the mass, vasculitis, eosinophilic infiltration, and necrosis. These features can mimic vasculitis. In a retrospective study, 16 out of 2642 patients evaluated for eosinophilia were found to have lymphoma.^[3]^ Therefore, lymphoma should be considered an important differential diagnosis for patients with atypical clinical manifestations of EGPA.
